# Spectral Analysis of Heart Rate Variability: Time Window Matters

**DOI:** 10.3389/fneur.2019.00545

**Published:** 2019-05-29

**Authors:** Kai Li, Heinz Rüdiger, Tjalf Ziemssen

**Affiliations:** ^1^Autonomic and Neuroendocrinological Lab, Center of Clinical Neuroscience, University Hospital Carl Gustav Carus, Dresden University of Technology, Dresden, Germany; ^2^Department of Neurology, Beijing Hospital, National Center of Gerontology, Beijing, China; ^3^Department of Neurology, University Hospital Carl Gustav Carus, Dresden University of Technology, Dresden, Germany

**Keywords:** trigonometric regressive spectral analysis, fast fourier tranform (FFT), heart rate variability, multiple trigonometric regressive spectral analysis, long-term, short-term

## Abstract

Spectral analysis of heart rate variability (HRV) is a valuable tool for the assessment of cardiovascular autonomic function. Fast Fourier transform and autoregressive based spectral analysis are two most commonly used approaches for HRV analysis, while new techniques such as trigonometric regressive spectral (TRS) and wavelet transform have been developed. Short-term (on ECG of several minutes) and long-term (typically on ECG of 1–24 h) HRV analyses have different advantages and disadvantages. This article reviews the characteristics of spectral HRV studies using different lengths of time windows. Short-term HRV analysis is a convenient method for the estimation of autonomic status, and can track dynamic changes of cardiac autonomic function within minutes. Long-term HRV analysis is a stable tool for assessing autonomic function, describe the autonomic function change over hours or even longer time spans, and can reliably predict prognosis. The choice of appropriate time window is essential for research of autonomic function using spectral HRV analysis.

## Introduction

Heart rate variability (HRV) is the physiological phenomenon of variation in heart beats. Even in resting states, spontaneous fluctuations of the intervals between two successive heart beats occur. Spectral analysis of HRV is a non-invasive and easy-to-perform tool for evaluating cardiac autonomic activity (1). Two critical frequency domain parameters obtained from spectral analysis are widely used: low frequency (LF) power (0.04–0.15 Hz) represents both sympathetic and vagal influences; high frequency (HF) power (0.15–0.40 Hz) reflects the modulation of vagal tone. In addition, LF/HF ratio indicates the balance between sympathetic and vagal tones (2). HRV analysis has been widely used in numerous cohorts, and plays an important role in describing the patients' autonomic dysfunctions, tracking the natural fluctuations of autonomic function, evaluating the autonomic changes following various interventions, and predicting prognosis.

## Preprocessing of the ECG Data

Before spectral analysis for HRV, there are a series of preprocessing steps. The preprocessing procedures include sampling and digitizing, artifact identification, RR data editing, RR interval rejection, NN data sequence; for some methods (e.g., fast Fourier transform) interpolation and sampling of the tachogram are needed (2). It is noticeable that these preprocessing steps could influence the HRV analysis results.

Firstly, the device should have a sufficient sampling rate. A low sampling rate (< 200 Hz) can affect the identification of QRS complex, and lead to inaccurate RR intervals, then leads to distorted HRV analysis (2, 3). It is recommended that the sampling rate should not be lower than 250 Hz (3).

Before spectral analysis, we need to carefully inspect the ECG to identify potential artifacts, ectopic beats, and arrhythmic events. Since HRV analysis is based on the sinus rhythm, if left untreated, these artifacts and non-sinus events would introduce errors (4). For short-term HRV analysis, if possible, recordings that are free of artifacts, ectopic beats, and arrhythmia should be chosen. If the selected data include technical artifacts, such as missed beats (caused by failure to detect the R peak) and electrical noise, we can edit the data by a proper interpolation based on the neighboring RR intervals (4). In contrast, the methods editing ectopic beats are uncertain. There are various approaches to mitigate the influence of ectopic beats, including deletions of the ectopic beats and numerous interpolation methods (5). Overall, simply deleting the ectopic beats is not recommended because it loses ECG information and obtains distorted LF and HF power (5, 6). The choice of interpolation methods depends on the type of ectopic beat, quality of data, and the study population. For a ventricular premature beat, the period between the normal beats before and after the premature beat is approximately twice the mean RRI, and an intermediate insertion between the two neighboring normal beats is acceptable (4, 7). However, a supraventricular ectopic beat can reset the sinoatrial node activity, and more complicated interpolation approaches might be appropriate (7). In this occasion, commonly used interpolation methods include interpolation of degree zero, interpolation of degree one, cubic spline interpolation, integral pulse frequency modulation model, etc. Until now, how to choose the best technique still needs further investigation, and head-to-head comparisons are warranted (5–7).

For fast Fourier transform, to satisfy the requirement of equal distance, interpolation is needed, and we would discuss this issue in the following section. Sometimes, automatic filters are employed; it might theoretically improve the statistical reliability of the data. But we should observe its impact to the spectral components, because this operation may incur errors (2, 4).

## Commonly Used Spectral HRV Analysis Methods

Most commonly, power spectral analysis of HRV is analyzed through fast Fourier transform and autoregressive models, by commercial devices or non-commercial software (8). In most cases, both methods obtain comparable results, but we need to notice their differences.

The algorithm of fast Fourier transform is relatively simple and has low computational cost. However, fast Fourier transform based spectral analysis is subjected to the problem of non-equal distance of RR intervals and a requirement of stationary data segments. In addition, the length of data segments influences the basic oscillation and the frequency resolution of fast Fourier transform analysis (2, 9). Therefore, fast Fourier transform based HRV analysis needs artificial interpolation to satisfy the demand on equal distance, but the interpolation would introduce biases. Typically, it works on a stable ECG segment of at least 5 min, this restriction on length sometimes limits its application (such as in dynamic processes) (2, 9).

The autoregressive method is also a popular tool for spectral analysis of HRV, it does not need interpolation, and the length of data required for analysis is shorter than fast Fourier transform. However, one of the disadvantages of the autoregressive method is its complexity, the choice of models and model order varies across different studies and this parameter substantially affects the results (2). Furthermore, several studies showed that the autoregressive method was not able to detect frequency domain parameters and generated null values in a substantial proportion of patients with diabetes or hypertension (10, 11).

There have been several autoregressive models in ECG signal processing. Burg's algorithm, the least square approach and Yule-Walker method are commonly used (12–14). Each method has its advantages and disadvantages. Both Yule-Walker method and Burg algorithm suffer from the problems of spectral line splitting and the bias in the positioning of spectral peaks (12, 14). However, Burg's algorithm has a better resolution and a higher spectral fidelity for short data records than Yule-Walker method, and Burg's algorithm has no implied windowing which distorts spectrum in Yule-Walker method (14, 15). The least square approach has improvements in the issues of spectral line splitting and the bias in the positioning of spectral peaks, but is less stable than Burg's algorithm (12, 13, 15). Generally, they obtain similar results in most situations (13, 16), but Burg's algorithm is a more stable approach and is preferable among the three methods (13, 15).

If the model order is too high, the model is more susceptible to the interference of noise and might slit peaks. If the model order is too low, the spectral peaks are smoothed considerably, their positions might be altered and some peaks might be missed; moreover, the analysis may even obtain null results (4, 17). There are many methods for the guidance of choosing the most appropriate order, such as Akaike information criteria, Akaike's final prediction error, final prediction error, Rissanen's minimum description length, etc. Akaike information criteria is the most widely used method (2, 4, 17).

Trigonometric regressive spectral (TRS) analysis is a newly developed and advanced analytical and statistical technique, it describes the rhythms of R–R intervals with trigonometric regression functions (18). In contrast to the fast Fourier transform, TRS does not need interpolation on non-equidistant heart beats, and provides a pure physiological spectrum using trigonometric regression. TRS searches one frequency at a time; therefore, the length of the data segment can be as short as 20–30 s (9, 18, 19). This feature makes TRS suitable for describing dynamic processes. TRS works in a shifting approach. Each analysis (TRS) spectrum is only performed within a local data segment (20–30 s); analyses of local data segments are repeated in successive segments shifted by one, two, or more beats within the whole global data segment (multiple TRS analysis, so called MTRS) (18, 19). Since the traditionally used global data segment is 1–2 min, we call this approach short-term MTRS. We have successively applied short-term MTRS analysis in healthy subjects and patients with various disorders (including diabetes and hypertension); it has been used for describing the resting cardiovascular autonomic function of specific populations and depicting the dynamic response of HRV to various stimulations, including Valsalva maneuver, metronomic deep breathing, head-up tilt test, cold pressor test, mental stress, and short-acting vasoactive medications (20–27). Overall, TRS is an outstanding technique for spectral analyses in autonomic function research in comparison to fast Fourier transform and the autoregressive method. We summarize the character of the three solutions for spectral analysis of HRV in Table 1.

**Table 1:** Summary of main spectral analysis methods.

**Methods**	**First publication on spectral analysis of HRV in human**	**Requirements for applications**	**Advantages**	**Disadvantages**	**Commonly used length in short-term spectral analysis**
FFT	•Sayers (28) •Hyndman et al. (29)	•Stationary ECG data •Sufficient length of data, •Equidistance between RRIs	•Simplicity of the algorithm, •High processing speed, •Good reproducibility •Widely available in commercial devices and research toolboxes	•Require interpolation, •Not appropriate for non-stationary data, •Need to work on an adequate length of data (usually 5 min), •Spectral components influenced by data length,	2–5 min; 5 min is preferred
Autoregressive models	•Pagani et al. (30, 31)	•Stationary ECG data	•Smoother spectral components, •Easy post-processing of the spectrum, •Lower requirements on the length of data than FFT, •Also widely available in commercial devices and research toolboxes	•Not appropriate for non-stationary data, •Complexity in choosing the suitable models, thus lack comparability between studies.	200–512 RRIs
MTRS	•Rudiger et al. (18)	•Only general requirements for HRV analysis such as free of ectopic beats and arrhythmia	•Can work on relatively short data segments (20–30 s), •Can be applied in non-stationary conditions, •Do not need interpolation and capture real physiological oscillations	•Relatively less widely available	1–5 min; 1.5–2 min is most frequently chosen

*ECG, electrocardiography; FFT, fast Fourier transform; HRV, heart rate variability; MTRS, multiple trigonometric regressive spectral analysis; RRI, RR interval*.

In addition, Lomb periodogram is another option for spectral analysis of HRV without the need of interpolation on RRIs. Lomb periodogram determines the power spectrum at any given frequency by fitting the sine wave using a least squares method. It outperformed fast Fourier transform and autoregressive method in several studies (32, 33). However, the non-random components of HRV and the 1/f noise in the spectra negatively influence the performance of Lomb periodogram (34). A recent study indicated that a smoothing procedure for Lomb periodogram may improve its capability in spectral analysis (35). Overall, Lomb periodogram is an relatively infrequently used method for spectral analysis, further methodological improvements and applications in clinical research are warranted (35, 36).

Furthermore, it is noteworthy that commonly used spectral analyses of HRV employ second order statistics, which is suitable for Gaussian distribution and linear systems (37, 38). However, the human cardiovascular system is not a linear system, and may not accord with Gaussian distribution. Theoretically, bispectral analysis has been developed to solve this problem (37, 38). Although studies using bispectral analysis for HRV assessment is still rare, it is a promising tool in HRV analysis. Since this article focuses on spectral analysis, further discussion of this method is out of the scope of this article.

## The Issue of Time Window in Spectral HRV Analysis

The time window of ECG analyzed is a key issue in the spectral analysis of HRV (39, 40). Most of the studies using spectral analysis of HRV via fast Fourier transform or autoregressive method work on ECG segments of 2–5 min, and previous applications of MTRS are on ECG segments of 1–2 min. Recently, various techniques for time-frequency analysis in non-stationary conditions have been developed, which mainly include short time Fourier transform, time variant autoregressive modeling, wavelet transform, and Wigner-Ville transform (41, 42). These techniques can obtain instant power spectral profiles of HRV during highly dynamic processes. Spectral analysis of HRV using longer time windows (usually from 1–24 h) has been reported, mainly using fast Fourier transform or autoregressive method. Long-term spectral analysis of HRV has been used in determining the autonomic function, assessing its changes, and predicting prognosis. Shorter and longer time windows have their own advantages and disadvantages according to the particular application scenarios. In the following sections, we will discuss the characteristics of short-term and long-term HRV analysis. In addition, we will also introduce our newly developed long-term MTRS analysis.

## Long-Term Spectral HRV Analysis Based on Short-Term Spectral HRV Analysis

**Figure 1:**
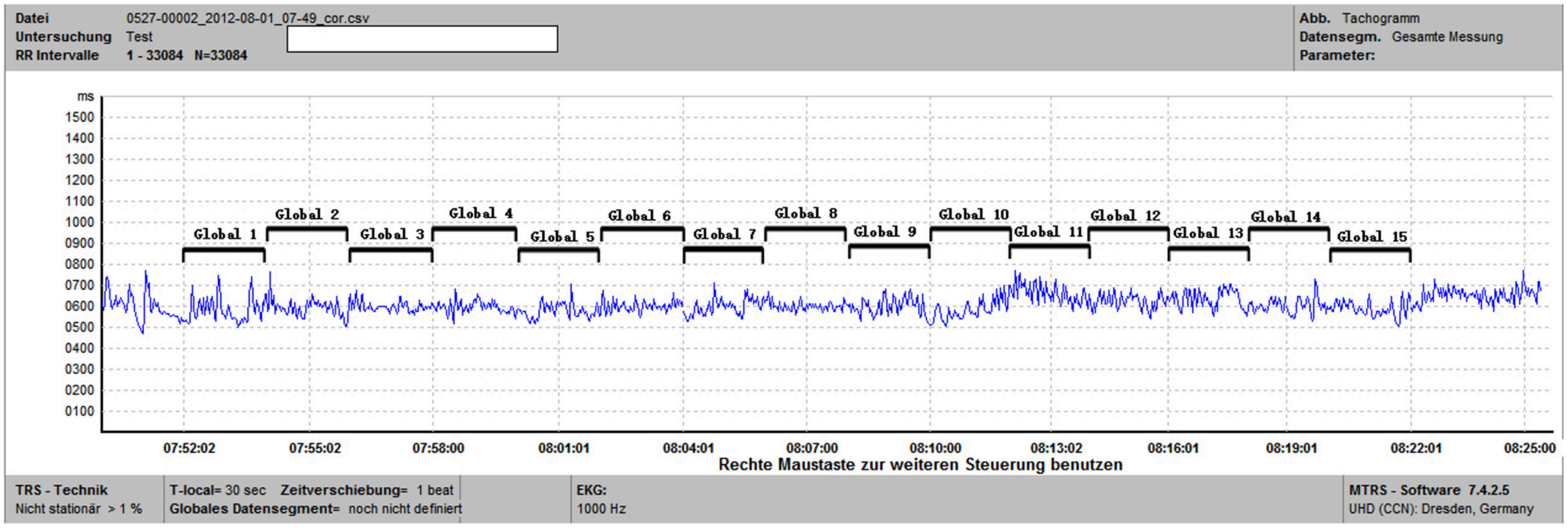
This is a tachogram from the MTRS software. For the specified time window of 30 min (from 07:52 to 08:22), we calculated the frequency domain parameters of 15 2-min global data segments (Global 1 to Global 15) and then computed the average of these 15 segments.

**Figure 2:**
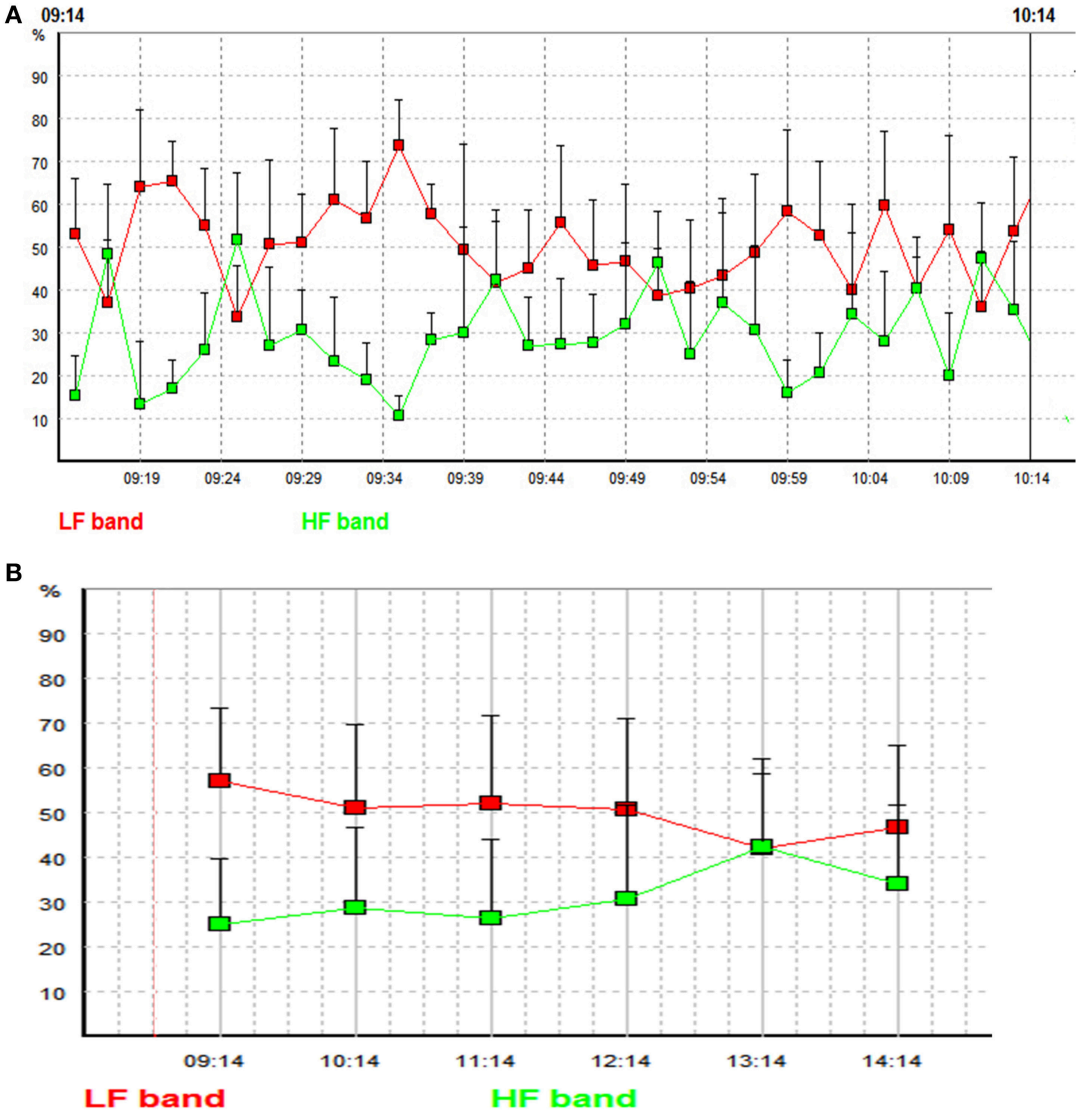
**(A)** LF and HF powers of the 30 2-min global segments within an hour (from 9:14 to 10:14 in the morning) in a patient with multiple sclerosis. Each square indicates the LF or the HF power of an individual 2-min global segment. Red line represents low frequency band, and green line represents high frequency band. The x-axis represents time and the y axis represents the relative LF and HF powers (the proportions (in percent) of LF and HF powers in the total power). **(B)** Mean LF and HF powers of the 6 h after fingolimod intake in a patient with multiple sclerosis. Each square indicates the mean value of LF or HF power of a targeted 1 h time window. Red line represents low frequency band, and green line represents high frequency band. The x-axis represents time and the y axis represents the relative LF and HF powers [the proportions (in percent) of LF and HF powers in the total power].

Fast Fourier transform and autoregressive based HRV analyses conventionally work on ECG recordings of 2–5 min (2, 3, 8). As mentioned earlier, short-term MTRS analysis mainly works on 1–2 min (2, 3). The short-term HRV analysis is often the basis for longer time windows. The most common strategy for long-term HRV analysis is to divide the target time window (e.g., 1 or 24 h) into consecutive 1–5 min epochs, and averaging the individual values of HRV parameters of all these epochs to obtain the mean value of the target time window (2, 39, 40). MTRS has been traditionally used in short-term HRV analysis, but recently we have developed a newer version of MTRS, which also used the averaging strategy. For a target ECG segment of 30 min to 24 h, this target ECG segment is firstly divided into consecutive 1–2 min global data segments. The spectral profiles of these consecutive 1–2 min global data segments are obtained through the shifting local data segments of 20–30 s as described before. Then the results of all the 1–2 min global data segments are averaged to obtain the mean values of the spectral parameters of the whole target time window. Figure 1 illustrates the strategy by showing how a given 30 min ECG recording is divided into 15 2-min global data segments. Each 2-min global segment was analyzed as our traditional short-term MTRS analysis, and then the results of all these 2-min global data segments are averaged to obtain the mean value of the whole targeted 30-min segment. This strategy can be applied even in longer time windows including 24 h. Figure 2A shows the LF and HF values of the 30 2-min global segments within an hour in a patient with multiple sclerosis, these 2-min values will be averaged to obtain the targeted 1-h results. Figure 2B shows an application of this long-term MTRS analysis in a patient with multiple sclerosis, who had taken 0.5 mg fingolimod. This figure shows the mean 1 h LF and HF powers of the 6 h after fingolimod intake. This dividing and averaging process is a common strategy for long-term spectral analysis of HRV, and the underlying algorithm can be fast Fourier transform, autoregressive method, or MTRS, etc. (43, 44).

Another strategy is to view the target time window as a whole data segment, and perform spectral analysis on this data segment en bloc (e.g., 1 or 24 h). For LF and HF, these two strategies obtained similar results over the 24 h time window (43).

## The Advantages and Disadvantages of Short and Long Time Windows

The cardiovascular system is a spatially and temporally complex system. It is built from a dynamic web of interconnected feedback loops. Heart rate, blood pressure, and HRV parameters keep fluctuating constantly, both in the resting state and under various internal and external stimulations (9, 45, 46). We can estimate HRV parameters in the resting state, during standing and daily activities, in different stages of sleep, and their responses to medications. Choosing the most appropriate time window for HRV analysis can optimize its application. Table 2 summarizes the advantages and disadvantages of short- and long-term spectral analysis of HRV.

**Table 2:** Advantages and disadvantages of short- and long-term spectral analysis of HRV.

	**Advantages**	**Disadvantages**
Short-term	•Easy to perform •Convenient to control the confounding factors •Needs less time for data processing •Can describe dynamic HRV change within a short period	•Not stable owing to the constant fluctuation of heart beat intervals •Cannot analyze ULF power
Long-term	•A stable tool for HRV analysis •Can analyze ULF power	•More expensive and time consuming •Include more noise •Influenced by activities and environmental factors

*HRV, heart rate variability; ULF, ultra-low frequency component*.

The advantages of short-term HRV analysis are as follows.

1. It is easy to perform; only several minutes' recording is enough.
2. It is convenient to control the confounding factors such as body position, physical activity, respiration, environmental factors like temperature.
3. It needs least time for data processing compared to long-term analysis.
4. Can describe dynamic HRV change within a short period.

Its main disadvantage is that the short-term HRV analysis might not be stable owing to the constant fluctuation of HRV parameters. Furthermore, short-term spectral analysis cannot estimate long RRI fluctuations, such as the ultra-low frequency component (ULF) (2, 39).

Time-frequency analysis can be viewed as a special case of short-term spectral analysis; it is able to closely track the instant changes of the spectral profiles of HRV. This is a fast developing area attracting active research. Short time Fourier transform and time variant autoregressive modeling are based on fast Fourier transform and autoregressive modeling; their time resolutions are not satisfactory (42). Wigner-Ville transform based time frequency-analysis has better temporal and frequency resolutions by independent controls of time and frequency filtering (47). However, its main limitation is the interference of cross-terms, which influences the accuracy. Various techniques seek to address this issue. Among them, smoothing in the time and frequency directions significantly suppresses the cross-terms, and smoothed pseudo Wigner–Ville distribution (SPWVD) have excellent performance in comparison with short time Fourier transform and time variant autoregressive modeling methods in comparative studies (42, 48). Wavelet transform also provides a good time resolution and the time-frequency resolution can be optimized by setting an appropriate wavelet filter. In contrast to short time Fourier transform, which has fixed windows for time and frequency, time resolution of wavelet transform depends on the frequency of interest and can obtain more precise spectral components (49). The limitations of wavelet analysis are: (1) the obtained frequency bands are not exactly the same as the recommendations of the Task Force of The European Society of Cardiology and The North American Society of Pacing and Electrophysiology. (2) The performance is unsatisfactory when more than one spectral component is present (49, 50). In several comparative studies, wavelet transform outperformed short time Fourier transform and time variant autoregressive modeling (42, 51, 52). Although mainly used in time-frequency analysis, wavelet transform has also been used in short-term spectral analysis, and was consistent with the output of fast Fourier transform (53). Other relatively less used time-frequency analyses include Gabor transform, modified B distribution, etc. Gabor transform can implement a signal adaptive analysis which eliminate the influence of noise and improves the accuracy (54, 55). Modified B distribution can achieve high time-frequency resolutions and suppress cross-terms (48). Time-frequency analysis is a fast developing field; further researches are needed to establish well-accepted techniques.

Long-term HRV analysis can collect ECG information from 1 h to an entire day. It is more stable than short-term analysis. Directly analyzing the entire long-term target time window can estimate longer fluctuations including the ULF power (2, 39). However, long-term recordings are more expensive and time-consuming; long-term recordings obtain more noise and dealing with the noise is challenging. In addition, environmental factors (temperature, humidity, etc.) and daily activities during recording vary within and between subjects.

To some extent, the comparison between short-term and 24-h long-term HRV analysis is similar as the comparison between office blood pressure and 24 h ambulatory blood pressure monitoring. Because the blood pressure also fluctuates constantly, office blood pressure has substantial randomness, while ambulatory blood pressure monitoring incorporates recordings during resting, activity, and sleeping of a whole day and is more stable than office blood pressure (56, 57).

## The Application of Spectral HRV Analysis in Different Occasions

It is noteworthy that age and gender can influence frequency domain HRV parameters. Generally, females have higher total power and HF power, and lower LF power and LF/HF ratio than males (40, 58–60). Some studies reported that this gender difference gradually disappear after the age of 40–50 (58, 59). With increasing age, total power and absolute values of LF and HF power decrease, while this trend is not significant for normalized values of LF and HF (22, 58–61). LF/HF ratio gradually increases until about 50 years, then decrease afterwards (60, 61). We should keep the effect of age and sex in mind in the application of spectral analysis of HRV.

### Evaluate Autonomic Function in a Specific Population

Short-term HRV analysis is frequently used in assessing the cardiovascular autonomic function of a specific population. Many diseases lead to a decreased HF power and/or an increased LF power, including patients with myocardial infarction (62), hypertension (63), cardiac syndrome X (64, 65), neurodegenerative diseases including Parkinson's disease, multiple system atrophy, and progressive supranuclear palsy (26, 66), affective disorders (67), and septic shock (68).

Long-term HRV analysis for this purpose is often performed on holter recordings, the researchers can select the daytime/awakening and the nighttime/sleep periods for analysis, or use the entire 24 h recording. In patients with panic disorder, the decrease of total power and ULF power is more pronounced during sleep than the whole day averaged values (69). In addition, HRV analysis of the recordings during sleep has shown that the patients with panic disorder had higher LF power than the controls, but the LF power during daytime or of the whole 20 h were similar in panic disorder patients and the controls (69). Haapaniemi et al. found that all the spectral components of HRV were lower in the patients with Parkinson's disease (70). Endurance-trained men have an increased HF power compared to untrained men over the 24 h (71).

Although in general, HRV analyses based on different lengths of time windows are closely correlated (72, 73), the results from different time windows are sometimes inconsistent. In the study by Yeragani et al. HRV analysis of the sleeping hours showed a higher relative LF power in the patients with panic disorder, which was not found for the awakening hours or the entire 24 h recordings (69). A study using short-term HRV analysis revealed an increased LF power in panic disorder during awakening time (67).

Considering the constant fluctuations of cardiovascular autonomic function, long-term HRV parameters may be more stable for describing the autonomic function in a specific population. If feasible, long-term ECG recordings of several hours to 24 h should be preferred over short-term ECG recordings for the assessment of autonomic state of a specific population.

### Description of Changes of Cardiac Autonomic Function

Both short and long time windows have been applied in evaluating changes across hours or months. In addition, short-term HRV analysis is able to track dynamic changes within a short time period such as several minutes.

Short-term HRV analysis has been widely used in tracking changes. It can measure real-time cardiac autonomic alterations during interventions. Thayer et al. computed 3.5–5 min ECG recordings during baseline, relaxation, and worry states. They found that worry was associated with decreased HF power (74). With short-term MTRS, Friedrich et al. assessed HRV changes from the resting state to deep breathing, orthostasis, and Valsalva maneuver in healthy controls, patients with Parkinson's disease, multiple system atrophy, or progressive supranuclear palsy, and found the different responses in the autonomic examinations of these distinct diseases (21). Short-term HRV analysis has also been used to measure changes over hours, such as circadian rhythm, changes during an acute disease course, and response after a medication. For this application, data segments of several minutes were selected from each hour, and then the HRV variables of the selected data segments were calculated as the representative results of the corresponding hours. This approach has been used to measure the circadian rhythm of HRV parameters in healthy subjects and shift workers, as well as the abnormalities of the HRV circadian rhythm in patients with hypertension (75–77). In addition to circadian changes, short-term HRV analysis has been used to delineate the cardiac autonomic function changes within several hours after taking a medication or other interventions, including levodopa, fingolimod, alcohol, and coronary angioplasty (78–81). Comparing resting short-term HRV parameters at baseline with those parameters several weeks, months, or even years later, it could reflect the changes of cardiac autonomic function across a long period. We have conducted a study which followed up patients with Wilson's disease for 3 years. We applied short-term MTRS analysis on ECG and blood pressure recordings during rest, deep breathing, orthostasis, and handgrip test. The short-term MTRS analysis on various examinations showed that cardiovascular autonomic function in Wilson's disease was generally stable across 3 years, though there were mild changes in a few parameters (79).

The remarkable ability to track instant changes of HRV makes time-frequency analysis a rapidly expanding field of research. Especially SPWVD and wavelet transform have an excellent temporal resolution, and have been used in delineating transient changes of HRV. In a study by Jasson et al., SPWVD continuously followed the changes of LF and HF during an orthostatic tilt test (52). In a recent study, SPWVD can be used to detect drowsiness by analyzing the HRV of three driving databases (82). Toledo et al. used wavelet transform to detect the instant change of autonomic tone during thrombolysis in myocardial infarction patients, at least one of the HRV parameters presented remarkable changes during the reperfusion process, and the infarct locations were related to the pattern of HRV parameter changes (83).

Long-term HRV analysis is commonly used in the studies of the circadian rhythm of cardiac autonomic function. In this type of studies, hourly HRV parameters (either averaging consecutive short segments within each hour or directly analyzing the whole hour en bloc) were usually calculated on selected hours of a 24 h holter recording. In addition, some studies computed the averaged daytime and nighttime averaged HRV parameters. In healthy subjects, LF power and LF/HF ratio have higher values during the daytime and lower values during the nighttime, while HF power has lower values during the daytime and higher values during the nighttime (84, 85). On the contrary, this circadian rhythm disappears in patients with acute myocardial infarction or ischemic stroke (84, 86). In addition to circadian rhythm, long-term HRV analysis can be used to measure the change of cardiac autonomic function caused by various interventions. Recently, we have applied our long-term MTRS HRV analysis in the investigation of the effects of fingolimod first dose on cardiovascular autonomic function. We calculated hourly HRV parameters using the long-term MTRS tool which employed an averaging approach as mentioned before. That study has revealed that the LF power decreased and HF power increased in the several hours after initial fingolimod administration, and these changes were consistent with heart rate alteration (40). Long-term HRV analysis integrating heart rate information of a whole day is a stable tool for the evaluation of the changes of cardiac autonomic function across long time spans. Initiating fingolimod treatment induces a transient alteration in cardiac autonomic function; its chronic effect has been disclosed by long-term HRV analysis. Simula et al. followed up the multiple sclerosis patients treated with fingolimod for 3 months. The long-term HRV analysis showed decreased absolute values of LF and HF powers compared with baseline, while the LF/HF ratio remained unchanged (58).

Generally, for changes across a long time, these HRV analyses based on different time windows yield similar results, such as two studies of HRV changes induced by fingolimod across several months in patients with multiple sclerosis, one of the studies used a 10 min finger arterial blood pressure recording and the other study used 24 h Holter recording (87, 88). However, we need to consider the effects of time window length. Although both short-term and long-term HRV analysis using the TRS algorithm showed that fingolimod increased HF in patients with multiple sclerosis, only long-term MTRS analysis showed that the changes of LF and HF were consistent with other related HRV parameters. This comparison indicates that long-term HRV analysis is more stable and can acquire consistent results among related variables (40, 79). Again, longer time windows obtain more stable results than short time windows. Thus, for tracking changes of cardiovascular autonomic function across minutes, only short-term HRV analysis can be used, while long-term HRV analysis should be preferred for depicting changes across hours to even longer time span if feasible.

### Predict the Patients' Outcomes

Initially, only long-term HRV parameters were used for the prediction of prognosis in patients with cardiovascular diseases (89). Then gradually, a variety of time windows have been applied in predicting the outcome in diverse diseases.

Short-term HRV analysis has been used in predicting outcomes both in the short-run and the long-run. Pontet et al. have identified LF calculated from a 10 min ECG as a good predictor of multiple organ dysfunction syndrome in the next few days in septic patients (90). In another study on sepsis patients, normalized HF power and standard deviation of the NN interval (SDNN) were valuable predictors of in-hospital mortality (91). For outcome prediction in the long-run, reduced short-term LF power during controlled respiration is a strong predictor of sudden death in the patients with chronic heart failure (92), and multiple time and frequency domain parameters obtained from a 2-min ECG recordings could predict end-stage renal disease and chronic kidney disease related hospitalization in the participants of the Atherosclerosis Risk in Communities (ARIC) study (93).

Because of its stability, long-term HRV analysis has been widely applied in the prediction of outcomes. Tsuji et al. analyzed the 2 h of ambulatory ECG recordings of the subjects of the Framingham Heart Study. After a 4-year follow-up, SDNN, and all the frequency domain parameters were associated with mortality, especially LF was the strongest predictor in a multivariate model (94). Long-term HRV analysis of 24 h ECG recordings performed in the acute, subacute, and chronic stages of myocardial infarction can predict mortality (44, 95, 96). Twenty-four-hour long-term HRV analysis has also been used to predict prognosis in patients with other cardiovascular diseases such as sudden cardiac death and idiopathic dilated cardiomyopathy (97, 98).

In addition to traditional frequency domain parameters, power law relationship of HRV is a derivative approach of long-term spectral HRV analysis. It describes the distribution of the spectral characteristics of RRI oscillations, and is computed by regressing the log (power) on the log (frequency) of HRV between frequencies 10^−2^ and 10^−4^ (99–101). The slope of the regression line is the most commonly used power law parameter for risk stratification (101, 102). The slope of the power law relation outperformed traditional long-term frequency domain HRV parameters in patients with acute myocardial infarction and elderly people (101, 102). Its performance in other populations warrants further investigation.

Comparing the predicting performance of different time windows is a critical issue, and previous studies have produced inconsistent results. Lü et al. demonstrated that SDNN of a 5-min ECG recording could predict the mortality of patients with myocardial infarction, but was inferior to long-term HRV indices (103). In contrast, Bigger et al. compared the power spectral measures of HRV calculated from short ECG recording segments (2, 5, 10, and 15 min) with those from 24 h long-term recordings, and concluded that both short and long-term HRV analyses were excellent predictors of mortality in patients with myocardial infarction (104). Malik et al. showed that HRV analysis of 24 h predicted prognosis better than HRV analysis based on arbitrary selected 1 h ECG segments in patients with myocardial infarction (105), while Voss et al. found that HRV analysis calculated from stationary daytime and nighttime 30 min ECG recordings provided at least a comparable prognosis prediction as 24 h long-term analysis (106).

Although these results seem incompatible, we need to note their methodological differences. Lü only used time domain parameters and other studies used frequency domain with or without time domain parameters. Voss et al. selected the most stationary daytime and nighttime 30 min ECG recordings, while some other studies chose the segments arbitrarily. We can refer to the researches on the abilities of office blood pressure and ambulatory blood pressure monitoring to predict prognosis. Ambulatory blood pressure monitoring predicts cardiovascular outcomes better than office blood pressure (57, 107). Similarly, long-term HRV analysis should be preferred for prediction of prognosis if applicable.

## Summary

In medical research, HRV analysis has been used to describe the autonomic function of specific populations, track the change of autonomic function across various time spans, and predict the patients' outcome. Short-term HRV analysis is easy to perform, with the prominent advantage that it can track dynamic changes of cardiac autonomic function within minutes. Recently developed time-frequency analysis further enhances the ability of HRV analysis to track active changes of cardiovascular autonomic function. The most predominant advantage of long-term HRV analysis is its stability, it is a stable tool for assessing the autonomic function, describe the chronic autonomic function changes over hours or even longer time spans, and can reliably predict prognosis in patients with cardiovascular diseases.

## Author Contributions

TZ, KL, and HR were involved in the conception and design of this article. KL performed the literature search and drafted the manuscript. TZ and HR critically revised the manuscript.

### Conflict of Interest Statement

The authors declare that the research was conducted in the absence of any commercial or financial relationships that could be construed as a potential conflict of interest.
